# Multilevel structure–activity profiling reveals multiple green tea compound families that each modulate ubiquitin-activating enzyme and ubiquitination by a distinct mechanism

**DOI:** 10.1038/s41598-019-48888-6

**Published:** 2019-09-05

**Authors:** Gabriel Fenteany, Paras Gaur, Lili Hegedűs, Kata Dudás, Ernő Kiss, Edit Wéber, László Hackler, Tamás Martinek, László G. Puskás, Lajos Haracska

**Affiliations:** 1 HCEMM-BRC Mutagenesis and Carcinogenesis Research Group, Institute of Genetics, Biological Research Centre of the Hungarian Academy of Sciences, 6726 Szeged, Hungary; 2 Department of Medical Chemistry, University of Szeged, 6720 Szeged, Hungary; 3 AstridBio Technologies Ltd., 6726 Szeged, Hungary; 4 Laboratory of Functional Genomics, Biological Research Centre of the Hungarian Academy of Sciences, 6726 Szeged, Hungary; 5 Avicor Ltd., 6726 Szeged, Hungary

**Keywords:** Screening, Mechanism of action, Ubiquitylation

## Abstract

We developed and implemented a reconstituted system to screen for modulators of the ubiquitination of proliferating cell nuclear antigen, a process that activates pathways of DNA damage tolerance and drug resistance. We identified the primary putatively health-beneficial green tea polyphenol epigallocatechin gallate (EGCG) and certain related small molecules as potent inhibitors of ubiquitination. EGCG directly and reversibly targets the ubiquitin-activating enzyme Uba1, blocking formation of the Uba1~ubiquitin thioester conjugate and thus ubiquitination and in the cell. Structure–activity relationship profiles across multiple biochemical and cellular assays for a battery of EGCG analogues revealed distinct chemical and mechanism-of-action clusters of molecules, with catechin gallates, alkyl gallates, and myricetin potently inhibiting ubiquitination. This study defines a number of related though distinct first-in-class inhibitors of ubiquitination, each series with its own unique activity pattern and mechanistic signature.

## Introduction

Substantial compelling evidence suggests inhibition of the DNA repair and damage tolerance machinery is a viable strategy for the development of therapeutics against cancers and other diseases. We are interested in discovering and leveraging small molecules that modulate the function of proteins involved in the DNA damage response as potential drug leads and research probes. Such agents would ideally limit the proliferation of and induce apoptotic death in diseased cells—either as standalone drugs or in synergistic combination with other treatments, such as chemotherapy, radiotherapy, and targeted therapies—and reverse loss of efficacy in cases of therapeutic resistance. The primary modes of DNA damage tolerance are the translesion synthesis and template switching pathways, which are engaged when replication forks stall at sites of unrepaired DNA damage and are unable to copy through the DNA (reviewed in^1–4^). These pathways involve transient changes in components of the replication machinery, allowing for replication past DNA lesions, though at a price: Translesion synthesis occurs through specialized polymerases that are inherently prone to error, whereas template switching can result in chromosomal rearrangements. DNA damage tolerance can thus lead to heightened mutagenesis, genomic instability, oncogenesis, formation of secondary tumors in cancer patients after treatment with DNA-damaging therapeutics, intrinsic and acquired drug resistance, and other pathologies (reviewed in^5–7^).

A principal activating step in DNA damage tolerance pathways is the modification of proliferating cell nuclear antigen (PCNA), a central replication and repair factor that forms a homotrimeric clamp encircling DNA, by covalent attachment of ubiquitin to PCNA. Ubiquitination of PCNA prerequires the ATP-dependent loading of PCNA onto DNA by replication factor C (RFC), which makes PCNA a competent substrate for efficient ubiquitination. The reaction cascade then proceeds via: (1) ATP-dependent activation of the carboxy terminus of ubiquitin catalyzed by the E1 ubiquitin-activating enzyme Uba1 (also known as UBE1) through formation of a ubiquitin adenylate intermediate that then reacts with the active-site cysteine on Uba1, yielding a high-energy Uba1~ubiquitin thioester intermediate; (2) transfer of the ubiquitin moiety to the catalytic cysteine on the E2 ubiquitin-conjugating enzyme Rad6 to form a Rad6~ubiquitin thioester intermediate; and (3) transfer of ubiquitin from Rad6 specifically to the side-chain amine of the K164 residue on PCNA to form a PCNA–ubiquitin isopeptide bond, mediated by the E3 ubiquitin ligase Rad18 in complex with Rad6 (reviewed in^1–4,8^).

We seek to identify and exploit new small-molecule modulators of the PCNA ubiquitination cascade as potential therapeutics and research tools. Thus, we developed a robust, reliable, and sensitive high-throughput assay to quantitatively measure PCNA ubiquitination, based on amplified luminescent proximity homogeneous assay (Alpha) technology. The assay is reconstituted from all the individual purified proteins (RFC, Uba1, Rad6, Rad18, PCNA, and ubiquitin) that comprise the minimal necessary and sufficient components for the loading of PCNA onto DNA and its subsequent monoubiquitination. Over the course of screening a chemical library for compounds that affect PCNA ubiquitination, we identified the flavonoid (−)-epigallocatechin-3-gallate (EGCG) as a potent inhibitor of the reaction cascade. EGCG is the main polyphenol in green tea and has numerous reported activities, with compelling potential in cancer prevention and treatment (having been shown in previous studies to inhibit cell growth, induce apoptosis, increase sensitivity to other cytotoxic agents, and mitigate drug resistance), as well as in applications against various neurodegenerative, cardiovascular, immune, inflammatory, metabolic, microbial, and viral diseases (reviewed in^9–30^).

Investigating in further detail the mechanism of EGCG’s strong inhibition of PCNA ubiquitination, we determined that EGCG blocks formation of the Uba1~ubiquitin thioester conjugate and directly targets Uba1 itself and not just secondarily to interaction or reaction with ubiquitin. Uba1 is required for the initial activation of ubiquitin for almost all ubiquitination events, with a consequently wide range of biological effects in normal and pathological states such as cancers and neurodegenerative diseases (reviewed in^31–33^). We evaluated the biological relevance of Uba1 as a genuine target of EGCG in the cell. We discovered that human embryonic kidney (HEK) 293 cells overexpressing Uba1 have reduced sensitivity to the cytostatic and cytotoxic effects of EGCG relative to control cells, implying that EGCG binds Uba1 in the cell with functional effects on cell viability. Through comparative evaluation of a panel of EGCG analogues across multiple assays to establish combined structure–activity relationship (SAR) profiles, we noticed distinct subclasses of molecules, with clusters of different activity patterns that are clearly related to chemical features common to each grouping. In particular, three SAR clusters—catechin gallates, alkyl gallates, and myricetin—display inhibitory activity against PCNA ubiquitination and Uba1~ubiquitin thioester formation, and consistent cellular activity, though each with its own apparent mechanistic profile. Collectively, our results delineate pharmacophores that may serve in the potential development of more selective agents as tools for research and chemotherapeutics against cancers and other diseases.

## Results

### Development of a high-throughput reconstituted screening system to identify modulators of PCNA ubiquitination

**Figure 1:**
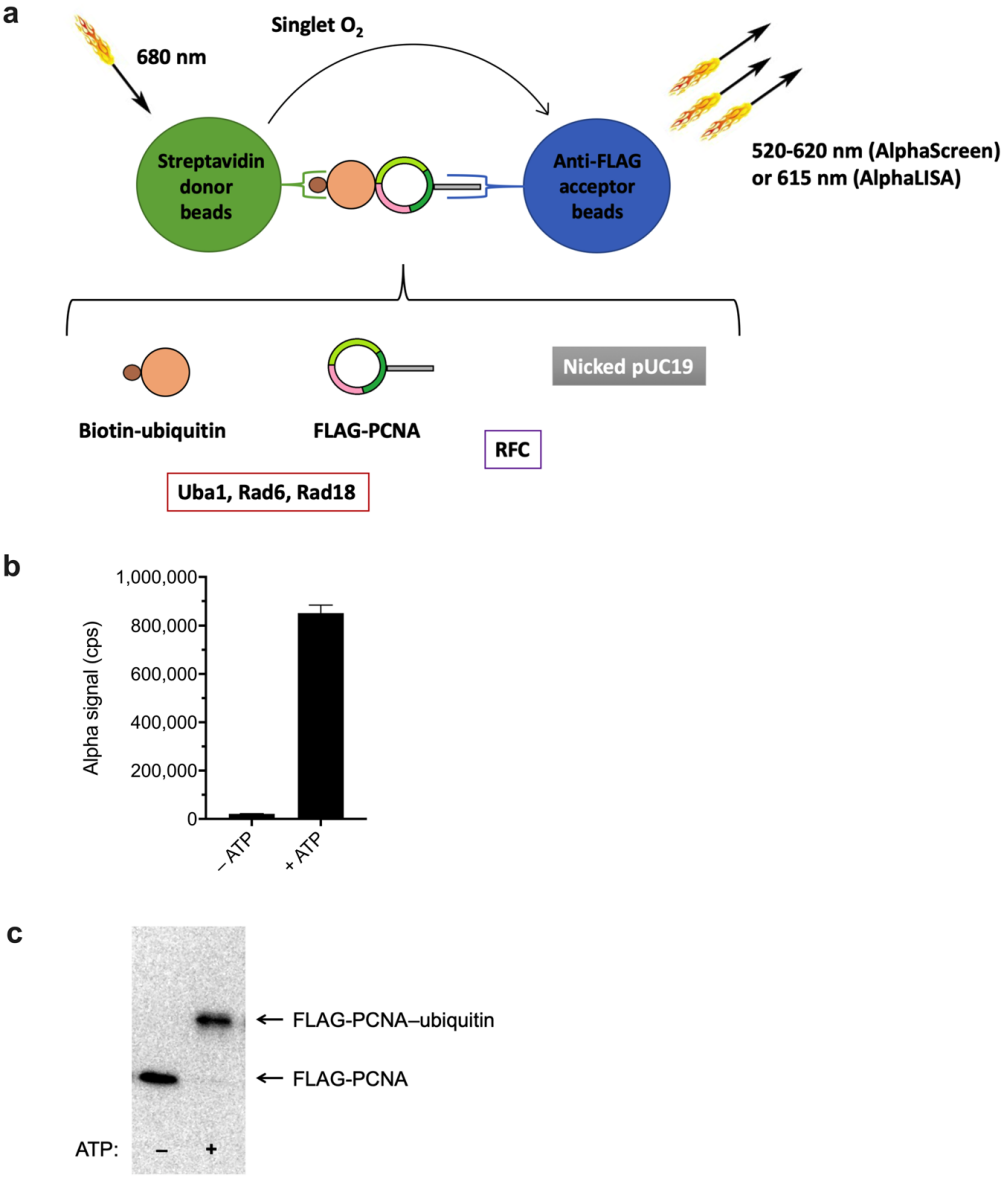
Reconstituted assay for PCNA ubiquitination. (**a**) High-throughput screening system for PCNA ubiquitination (FLAG-PCNA and biotin-ubiquitin proximity) *in vitro*, based on PerkinElmer’s AlphaScreen/AlphaLISA technology.  Note: Unlike in this simplified diagram, each of the three subunits of the PCNA trimer bears a FLAG tag and each PCNA molecule is monoubiquitinated on K164 at saturation in the reaction; thus, higher bead-to-trimer stoichiometry will occur than indicated (in principle, three donor and three acceptor beads per complex, depending on steric factors). (**b**) Quantitative measurement of PCNA ubiquitination by the Alpha system for non-initiated (−ATP) and initiated (+ATP) ubiquitination reaction cascades. Data represent mean and standard deviation (SD) for six independent experiments for Alpha signal values in counts per second (cps) following incubation without or with ATP for 2 h, under conditions described in Methods. (**c**) Representative Western blot of samples incubated without or with ATP for 2 h probed with anti-FLAG antibody, as described in Methods, showing nonubiquitinated FLAG-PCNA (lower band) and ubiquitinated FLAG-PCNA (upper band).

**Figure 2:**
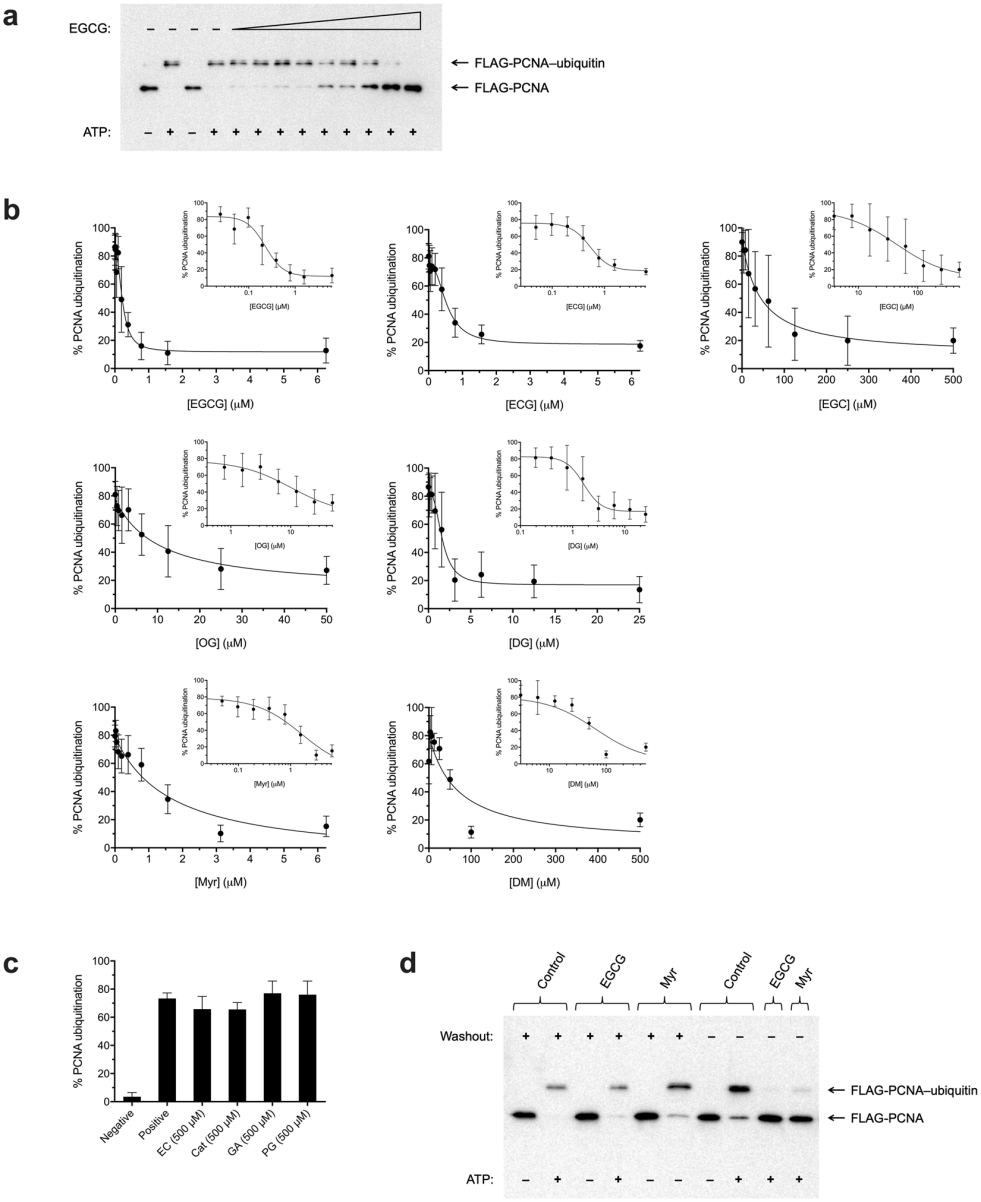
Catechin gallates, alkyl gallates, and myricetin inhibit PCNA ubiquitination. (**a**) Representative Western blot probed with anti-FLAG antibody showing the dose–response for inhibition of PCNA ubiquitination by EGCG (0.0122 to 6.25 μM in concentration increments of 2×), with a final DMSO carrier solvent concentration of 2% in all cases. Samples were preincubated with EGCG for 15 min prior to addition of ATP, then further incubated for 2 h. Negative and positive controls consisted of parallel samples incubated with DMSO alone in the absence (−) or presence (+) of ATP, respectively. (**b**) Dose–response for % PCNA ubiquitination for the different compounds, plotted both linearly and semi-logarithmically (inset), with a DMSO concentration of 2% in all cases. Data represent mean ± SD, derived from quantitation of Western blot images according to *(FLAG-PCNA–ubiquitin)/(FLAG-PCNA* + *FLAG-PCNA–ubiquitin)* × 100% for each lane internally from 3–7 independent experiments, as indicated for each case in Table 1 along with IC_50_ values. (**c**) % PCNA ubiquitination for compounds with no inhibitory activity even at 500 μM, with a DMSO concentration of 2% in all cases. Data represent mean and SD for three independent experiments. Negative and positive controls consisted of DMSO alone without or with ATP, respectively. (**d**) Effects of EGCG and Myr on Uba1 and ubiquitination are reversible. 30 μM EGCG or 30 μM Myr (with DMSO alone as control and a DMSO concentration of 2% in all cases) were preincubated with Uba1 for 2 h, completely washed out by serial centrifugal filtration, and then assayed for PCNA ubiquitination activity after addition of other reaction components, with detection by Western blot analysis with anti-FLAG antibody.

We designed and implemented a quantitative high-throughput assay for PCNA ubiquitination based on PerkinElmer’s AlphaScreen/AlphaLISA system (Fig. 1a), with wide dynamic range, and very strong signal in positive control relative to negative control (Fig. 1b), as also confirmed by Western blot analysis (Figs 1c and 2). We found the assay to be reliable, robust, and scalable. It consists of a reconstituted system containing biotin-conjugated ubiquitin, FLAG-tagged PCNA, nicked circular pUC19 plasmid, RFC, Uba1, Rad6, and Rad18, adapted from a previously published assay^34^, with ubiquitination of PCNA by Rad6–Rad18 being specific for the K164 residue, as K164R mutant PCNA protein is not ubiquitinated^34,35^.

Compounds were transferred to plates and briefly incubated to ensure equilibrium binding, then ATP was added to initiate the PCNA loading and ubiquitination reaction cascades. After incubation, we stopped the reactions by addition of EDTA to chelate Mg^2+^, diluted in buffer containing Alpha donor and acceptor beads, then loaded and read the plates in a microplate reader with attached stacker and dedicated laser for excitation of donor beads. We conducted each experiment in triplicate, with multiple positive and negative controls on each plate.

### EGCG inhibits PCNA ubiquitination

We identified EGCG (structure in Fig. 6) as one of the bioactive hits from a pilot screen of *ca*. 6,400 compounds. Dose–response experiments revealed that EGCG inhibits PCNA ubiquitination *in vitro* (Fig. 2a,b) with a calculated half-maximal inhibitory concentration (IC_50_) of 228 nM (Table 1).

### EGCG blocks Uba1~ubiquitin thioester formation

**Figure 3:**
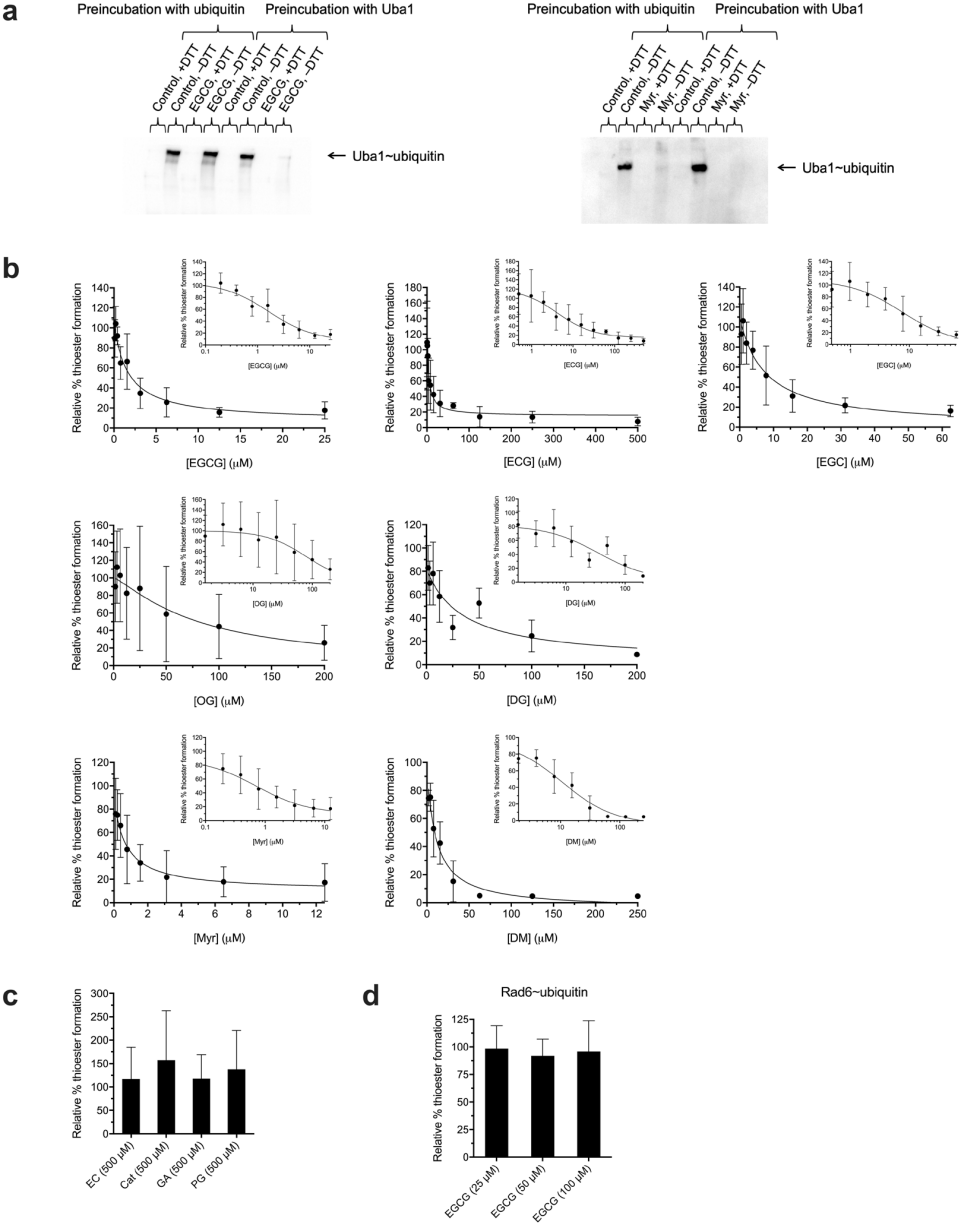
Catechin gallates, alkyl gallates, and myricetin inhibit Uba1~ubiquitin thioester formation. (**a**) Representative Western blots showing that EGCG inhibits Uba1~ubiquitin thioester formation when preincubated with Uba1 before adding ubiquitin, but not when preincubated with ubiquitin first, while Myr inhibits Uba1~ubiquitin thioester in both cases. Final compound concentrations were 10 μM for EGCG or 50 μM for Myr (with DMSO alone as control and a DMSO concentration of 2% in all cases). Following preincubation, reactions were allowed to proceed for 2 h with ubiquitin, Uba1, and ATP. Comparison of results in the absence to presence of DTT as reducing agent confirms that the conjugation is through the reducible thioester. (**b**) Dose–response for relative % Uba1~ubiquitin thioester formation with increasing concentrations of each compound, plotted both linearly and semi-logarithmically (inset), with a DMSO concentration of 2% in all cases. Data represent mean ± SD for 3–4 independent experiments, as indicated along with IC_50_ values for each case in Table 1. Each value was normalized on a gel-by-gel basis to the parallel positive control (DMSO alone with ATP) level of Uba1~ubiquitin thioester conjugate formed in each case and from quantitation of silver-stained SDS-polyacrylamide gels of reaction samples without DTT. (**c**) Relative % Uba1~ubiquitin thioester formation for compounds with no inhibitory activity even at 500 μM, with a DMSO concentration of 2% in all cases. Data represent mean and SD for four independent experiments. (**d**) EGCG does not inhibit formation of the Rad6~ubiquitin thioester conjugate resulting from transfer of ubiquitin from already formed Uba1~ubiquitin. The assay, with a DMSO concentration of 2% in all cases, and quantitation were done analogously to those for the Uba1~ubiquitin thioester formation experiments and are described in Methods. Data represent mean and SD for four independent experiments.

Inhibition of overall PCNA ubiquitination in our reconstituted system could be caused by direct inhibition of any of the components of the reaction sequence: Uba1, Rad6, Rad18, PCNA, or ubiquitin (or RFC, since loading of PCNA onto DNA is a requisite for its ubiquitination). We therefore looked at specific steps in the cascade and found that EGCG inhibits formation of the Uba1~ubiquitin thioester adduct (Fig. 3a,b and Table 1) in an assay consisting only of Uba1, ubiquitin, and ATP. Furthermore, preincubation of Uba1 alone before adding ubiquitin and ATP resulted in complete inhibition, whereas preincubation of ubiquitin alone with EGCG prior to adding Uba1 and ATP did not (Fig. 3a).

We conducted reversibility experiments by preincubating Uba1 or ubiquitin with EGCG and then washing out unbound compound by serial centrifugal filtration, followed by addition of other components to assay for ubiquitination. We found that ubiquitination activity recovers upon washout of compound, demonstrating that EGCG inhibits Uba1 and consequent ubiquitination in a reversible manner (Fig. 2d).

### Nuclear magnetic resonance (NMR) shows that EGCG binds Uba1

**Figure 4:**
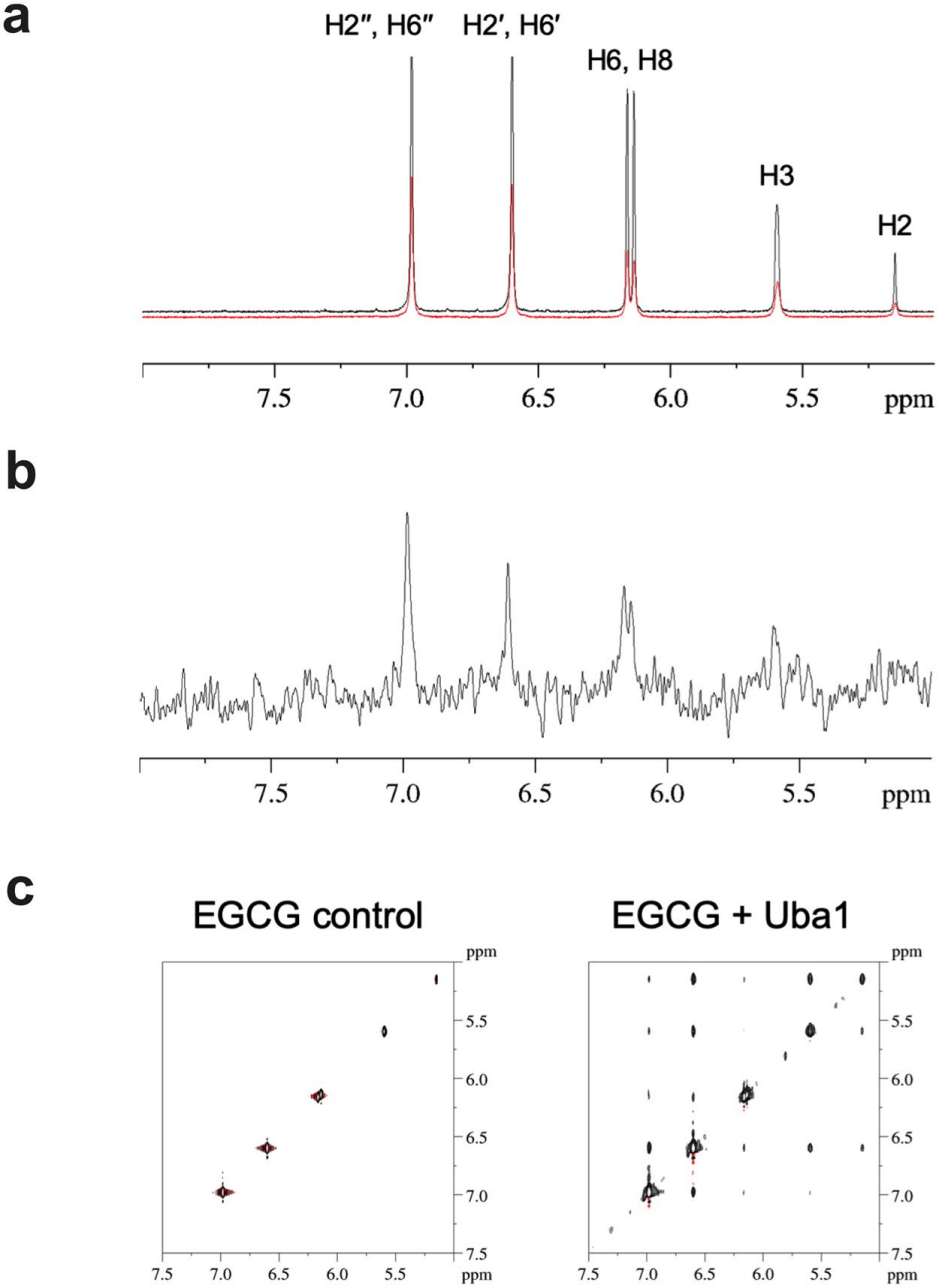
NMR reveals that EGCG directly binds Uba1. (**a**) Aromatic ^1^H NMR region of EGCG in the presence of Uba1 (red) and without protein (black). (**b**) STD NMR spectrum recorded for EGCG in the presence of Uba1, aromatic region. (**c**) TrNOE experiment: no crosspeaks are present in the EGCG control experiment (left), while several crosspeaks appeared in the presence of Uba1 (negative NOE due to an equilibrium between the free and the bound state of EGCG). The data suggest a *K*_d_ in the range of high nM to low μM, as discussed in Results.

The binding of EGCG to Uba1 was established by ligand-detected ^1^H NMR techniques. In the presence of Uba1, signal intensity loss was observed in the aromatic ^1^H NMR signals of EGCG compared with the control spectrum containing no protein (Fig. 4a), indicative of a dissociation constant (*K*_d_) of low micromolar or less. The interaction was also tested by standard saturation transfer difference (STD) NMR^36–38^ and transferred nuclear Overhauser effect (trNOE) experiments. In the STD spectrum of EGCG, ^1^H signals of EGCG were clearly detected, demonstrating ligand binding to Uba1 (Fig. 4b). The interaction was also confirmed by trNOE NMR. In presence of Uba1, positive crosspeaks were observed due to the higher cross relaxation rates of EGCG upon protein binding (Fig. 4c). In the 2D NOESY spectrum of the EGCG control sample, no crosspeaks appeared. Both STD and trNOE requires dynamic equilibrium between the free and the bound states of the ligand and are sensitive in the *K*_d_ range of 10^−3^–10^−8^ M. As signal intensity loss of EGCG signals was also observed, the NMR measurements suggest a *K*_d_ for the EGCG–Uba1 interaction in the high-nanomolar-to-low-micromolar range for a single binding mode.

### Overexpression of Uba1 or ubiquitin protects cells from EGCG’s cytostatic/cytotoxic effects and EGCG inhibits global cellular ubiquitination

**Figure 5:**
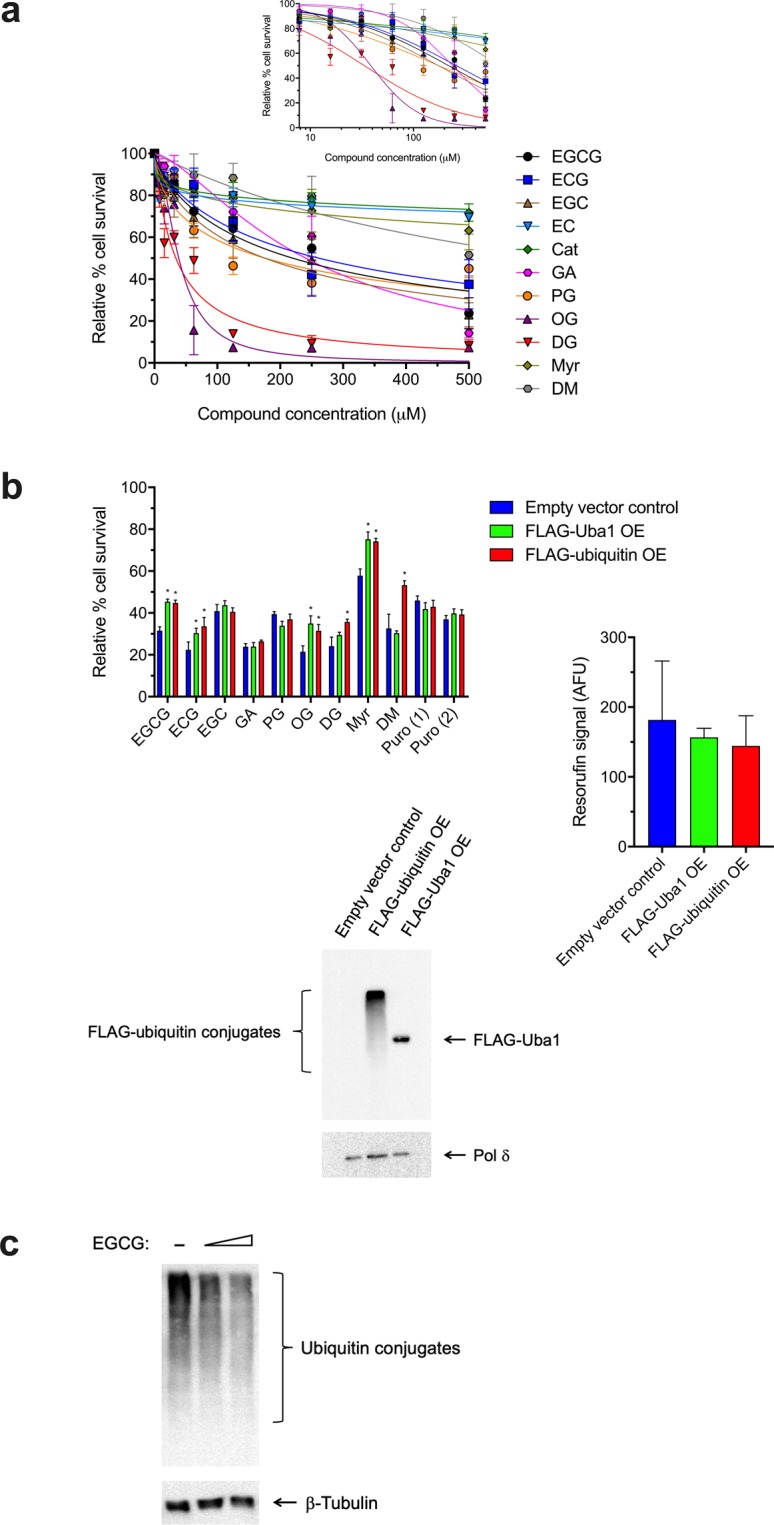
Catechin gallates, alkyl gallates, and myricetin reduce cell viability in a manner protected by overexpression of Uba1 or ubiquitin in cells and EGCG inhibits global cellular ubiquitination. (**a**) Dose–response for effects on HEK 293 cell survival after 24-h treatment with each compound relative to parallel untreated controls by the Alamar Blue (resazurin) assay. Data represent mean ± SD for 4–6 independent experiments (as indicated along with IC_50_ values for each case in Table 1), plotted both linearly and semi-logarithmically (inset), with a DMSO concentration of 2% in all cases. (**b**) Overexpression of Uba1 or ubiquitin reduces sensitivity of cells to the cytostatic/cytotoxic effects of the compounds that also have *in vitro* activity against PCNA ubiquitination and Uba1~ubiquitin thioester formation (Figs 2b and 3b) but not those without activity (Figs 2c and 3c). Data represent mean and SD for % cell survival relative to parallel controls (DMSO alone) for three independent experiments, with a DMSO concentration of 2% in all cases. Higher survival values following compound treatment between Uba1- or ubiquitin-overexpressing HEK 293 cells compared to the corresponding empty vector-transfected control cells after 24-h compound treatment are indicated with an asterisk (*p* < 0.003 in all cases by unpaired two-tailed Student’s *t*-tests). Treatment concentrations were at the IC_50_ values initially calculated for each compound shown in Table 1 from the data in (**a**), as well as 1 μM and 2 μM for puromycin—denoted as “Puro (1)” and “Puro (2),” respectively—to evaluate effects of an unrelated cytotoxic agent. Note: EC, Cat, Myr, and DM all exhibited only very weak activity at 500 μM in the cell viability experiments in (**a**), and we were not able to test over high enough concentration range for proper determination of their IC_50_ values. However, of these four compounds, we chose to still examine whether Uba1 and/or ubiquitin overexpression affected cell survival for Myr at 3 mM and DM at 650 μM (corresponding to their crudely guesstimated IC_50_ values), respectively, because Myr and DM had *in vitro* activity (Figs 2b and 3b), whereas EC and Cat did not (Figs 2c and 3c). Western blots of lysates from cells transfected with empty vector control, FLAG-ubiquitin-expressing, or FLAG-Uba1-expressing vector probed with anti-FLAG antibody, then stripped and probed with an anti-DNA polymerase δ catalytic subunit antibody as a loading control. The graph at right shows that Uba1 or ubiquitin overexpression by themselves had no effect on cell number in the parallel control cells in DMSO alone (mean and SD for three independent experiments), expressed as resorufin product signal in arbitrary fluorescence units (AFU) resulting from the metabolic reduction of resazurin in the assay. OE = overexpression. (**c**) Representative Western blot showing inhibition of global ubiquitination by EGCG in HEK 293 cells. Cells were treated with EGCG at 250 μM and 500 μM (with DMSO alone as control and a DMSO concentration of 1% in all cases) for 30 min, then MG132 was added to 50 μM, and cells were incubated for another 30 min, for a total EGCG treatment time of 1 h. Equivalent loadings of total protein from whole-cell lysates were subjected to Western blot analysis with anti-ubiquitin antibody and then with anti-β-tubulin antibody as a housekeeping protein loading control.

Treatment with EGCG reduces the viability and growth of HEK 293FT cells (Fig. 5a). Transfected HEK 293 cells overexpressing FLAG-tagged Uba1 or FLAG-tagged ubiquitin exhibited reduced sensitivity to EGCG compared to cells transfected with FLAG-tag-containing empty vector alone (Fig. 5b). In contrast, the unrelated cytotoxic compound puromycin displayed no differences in apparent toxic potency between normal and Uba1- or ubiquitin-overexpressing cells (Fig. 5b). Moreover, EGCG treatment inhibited the accumulation of ubiquitin-conjugated protein species in response to treatment with the peptide aldehyde proteasome inhibitor MG132 (Fig. 5c).

### SAR with EGCG analogues reveals distinct groupings of congeners by structure, activity profile, and mechanisms of action

**Figure 6:**
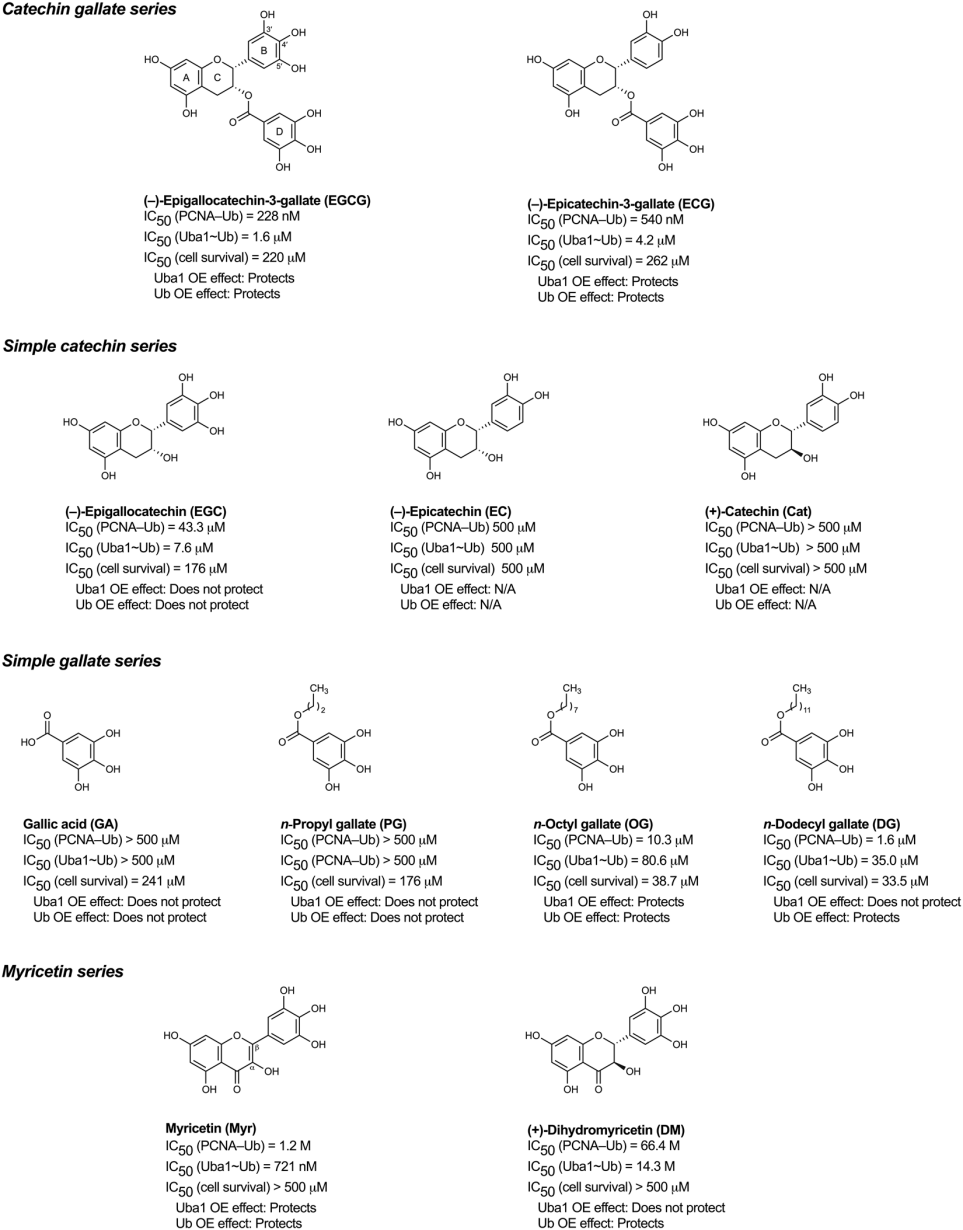
Structure–activity relationships for inhibition of PCNA ubiquitination, Uba1~ubiquitin thioester formation, and viability of normal, Uba1-overexpressing, and ubiquitin-overexpressing cells. More detailed information is contained in Figs 2, 3 and 5 and Table 1. OE = overexpression; N/A = not applicable.

We tested a series of EGCG-related molecules (structures in Fig. 6) for potential inhibitory activity in the same assays as before: PCNA ubiquitination *in vitro* (Fig. 2b,c), Uba1 charging with ubiquitin to form the Uba1~ubiquitin thioester adduct *in vitro* (Fig. 3b,c), and effects on the viability of HEK 293 cells (empty vector-transfected control, Uba1-overexpressing, and ubiquitin-overexpressing; Fig. 5b). All of the compounds tested have molecular weights (170.120–458.375) and predicted log*P* values (0.72–5.95, calculated with the Consensus model implemented in ChemAxon MarvinSketch 19.2) that suggest no likely questions of low cell permeability.

The combined SAR results from Figs 2, 3 and 5 are summarized with structures in Fig. 6, with calculated IC_50_ values for inhibition in the different assays presented in Table 1. EGCG was inhibitory in all the assays. The other catechin galloyl ester tested, (−)-epicatechin-3-gallate (ECG), was also active in all the assays (Figs 2b, 3b and 4a), and its inhibitory effect on cell viability was mitigated with overexpression of Uba1 or ubiquitin, as with EGCG (Fig. 5b). Of the unesterified catechins tested, (−)-epigallocatechin (EGC) displayed weak activity in the PCNA ubiquitination (Fig. 2b) and Uba1~ubiquitin thioester formation assays (Fig. 3b), while (−)-epicatechin (EC) and (+)-catechin (Cat) were inactive in both of these assays (Figs 2c and 3c). Unlike EC and Cat, EGC also has measurable cytostatic/cytotoxic activity (Fig. 5a). However, EGC’s negative effect on cell survival was not affected by Uba1 or ubiquitin overexpression (Fig. 5b). Free gallic acid (GA) and *n*-propyl gallate (PG), a short straight-chain alkyl galloyl ester, were inactive in the PCNA ubiquitination (Fig. 2c) and Uba1~ubiquitin thioester formation assays (Fig. 3c) but had little effects on cell viability (Fig. 5a). What inhibitory activity was observed was not mitigated with Uba1 or ubiquitin overexpression (Fig. 5b). Longer linear alkyl galloyl esters—*n*-octyl gallate (OG) and *n*-dodecyl (lauryl) gallate (DG)—were active in all the assays (Figs 2b, 3b and 4a), and overexpression of Uba1 or ubiquitin protected cells from their negative effects on cell survival (Fig. 5b). Myricetin (Myr) and (+)-dihydromyricetin (DM; also known as ampelopsin) were active against PCNA ubiquitination (Fig. 2b) and Uba1~ubiquitin thioester formation (Fig. 3a,c) but only had very weak activity against cell survival (Fig. 5a), although cells were protected from even that weak cytostatic/cytotoxic activity by overexpression of Uba1 or ubiquitin (Fig. 5b).

The alkyl gallates inhibited PCNA ubiquitination and Uba1~ubiquitin thioester formation with a potency directly proportional to the length of their alkyl chains in the rank order: DG > OG ≫ PG (the last one more-or-less inactive); Myr and DM also inhibited PCNA ubiquitination and Uba1~ubiquitin thioester formation in the order: Myr ≫ DM (Figs 2, 3 and 5 and Table 1). However, with Myr, unlike with EGCG, preincubation of not only Uba1 alone but also ubiquitin alone prior to adding the other reaction components for thioester formation resulted in inhibition (Fig. 3a). Since the presence of a Michael acceptor functionality in Myr makes a mechanism involving a covalent reaction with the target, which often but not always is irreversible, we tested whether Myr irreversibly inhibits PCNA ubiquitination or not by determining if enzyme activity recovers after washing out of compound through serial centrifugal filtration. Myr’s inhibitory effects on PCNA ubiquitination, like EGCG’s, were reversible (Fig. 2d).

**Table 1:** Half-maximal inhibitory concentration (IC_50_) values for compounds against PCNA ubiquitination and Uba1~ubiquitin thioester formation and effects on viability of normal, Uba1-overexpressing, and ubiquitin-overexpressing cells.

Compound	IC_50_ for inhibition of PCNA ubiquitination	IC_50_ for inhibition of Uba1~ubiquitin thioester formation	IC_50_ for inhibition of cell survival	Effect of Uba1 overexpression on cell survival	Effect of ubiquitin overexpression on cell survival
EGCG	0.2280 μM (SE = 0.03419; CI: 0.1714–0.3192; *n* = 7)	1.632 μM (SE = 0.4580; CI: 0.8348–3.451; *n* = 4)	220.2 μM (SE = 18.90; CI: 187.4–263.5; *n* = 6)	Protects	Protects
ECG	0.5369 μM (SE = 0.09460; CI: 0.3750–0.8431; *n* = 4)	4.223 μM (SE = 2.161; CI: 1.263–13.77; *n* = 6)	261.7 μM (SE = 33.88; CI: 206.2–353.4; *n* = 6)	Protects	Protects
EGC	43.28 μM (SE = 22.81; CI: 16.08–129.0; *n* = 4)	7.584 μM (SE = 3.869; CI: 2.927–20.67; *n* = 7)	175.7 μM (SE = 18.05; CI: 144.0–218.5; *n* = 4)	Does not protect	Does not protect
EC	>500 μM (*n* = 3)	>500 μM (*n* = 4)	>500 μM (*n* = 4)	N/A	N/A
Cat	>500 μM (*n* = 3)	>500 μM (*n* = 4)	>500 μM (*n* = 4)	N/A	N/A
GA	>500 μM (*n* = 3)	>500 μM (*n* = 4)	240.7 μM (SE = 18.12; CI: 206.6–280.3: *n* = 4)	Does not protect	Does not protect
PG	>500 μM (*n* = 3)	>500 μM (*n* = 4)	176.3 μM (SE = 23.55; CI: 135.8–240.4; *n* = 4)	Does not protect	Does not protect
OG	10.32 μM (SE = 6.406; CI: 3.182–40.09; *n* = 4)	80.60 μM (SE = 33.90; CI: 34.81–405.1; *n* = 4)	38.70 μM (SE = 3.106; CI: 32.72–45.46; *n* = 4)	Protects	Protects
DG	1.633 μM (SE = 0.3117; CI: 1.048–2.670; *n* = 5)	35.00 μM (SE = 25.11; CI: 6.288–533.1; *n* = 4)	33.46 μM (SE = 3.247; CI: 27.15–40.83; *n* = 4)	Does not protect	Protects
Myr	1.213 μM (SE = 0.2083; CI: 0.8755–4.201; *n* = 5)	0.7207 μM (SE = 0.4970; CI: 0.1766–2.680*; n* = 5)	>500 μM (*n* = 6)	Protects	Protects
DM	66.43 μM (SE = 38.92; CI: 27.21–180.9; *n* = 3)	14.27 μM (SE = 3.140; CI: 5.927–22.18; *n* = 3)	>500 μM (*n* = 6)	Does not protect	Protects

All IC_50_ values, standard errors, and 95% confidence intervals listed were calculated by nonlinear regression with GraphPad Prism software. The effects of compounds on cell survival in Uba1 and ubiquitin-overexpressing cells relative to empty vector-transfected control cells are also shown. SE = standard error; CI = 95% confidence interval; *n* = number of independent experiments; N/A = not applicable.

## Discussion

We have designed and implemented a powerful quantitative high-throughput assay for PCNA ubiquitination, a key early event in DNA damage tolerance processes (reviewed in^1–4,8^). The system is an *in vitro* reconstituted ubiquitination reaction cascade, based on the highly sensitive Alpha proximity-based luminescence assay system (Fig. 1a,b), with reliability confirmed by Western blot analysis (Figs 1c and 2). In the Alpha system, upon high-energy irradiation at 680 nm, a photosensitizer, phthalocyanine, embedded in the Alpha donor beads converts ambient ground-state oxygen to an excited singlet state (distinct from the far more reactive oxygen radical). Singlet oxygen diffuses away and reacts with a thioxene derivative in the Alpha acceptor beads (within a practical range of ~200 nm), producing a chemiluminescent emission that then excites fluors also present in the acceptor beads, yielding fluorescence detected by a photomultiplier tube. There are two general variants of the Alpha assay, the so-called AlphaScreen and AlphaLISA assays, differing only in the dyes downstream of thioxene in the formulation of the acceptor beads (anthracene and rubrene for AlphaScreen and a europium chelate for AlphaLISA), yielding different emission spectra maxima (520–620 nm for AlphaScreen or 615 nm for AlphaLISA acceptor beads), with all other aspects of the Alpha assay variants being the same. We found no difference in performance between the two variants in our system, where there are no components, as in serum or plasma samples, that might interfere with the AlphaScreen emission.

Screening for modulators of PCNA ubiquitination in a reconstituted system (consisting of the necessary and sufficient protein components of the cascade: RFC, Uba1, Rad6, Rad18, PCNA, and ubiquitin), we identified EGCG (structure in Fig. 6) as a potent inhibitor of ubiquitination (Fig. 2a,b). EGCG is a plant secondary metabolite, the most abundant polyphenol in green tea and thought to be its primary health-promoting agent. EGCG can bind and affect the activities of various proteins, with different degrees of potency, as well as function alternately as an antioxidant or a prooxidant; the compound possesses cytostatic and cytotoxic activity, can potentiate the effects of other treatments, and can reverse drug resistance in cells (reviewed in in^9–30^). Intriguingly, a defined green tea extract formulation (known as “sinecatechins”), composed of EGCG as the single largest component, is a United States Food and Drug Administration-approved topical treatment for human papillomavirus pathogenesis (reviewed in^39^), a process that is absolutely dependent on ubiquitination and degradation of p53, pRb, IκB, and other proteins involved in cell cycle checkpoint control (reviewed in^40,41^) and involves a virus-induced replication stress response hijacked for viral replication (reviewed in^42^).

We found that EGCG abrogates formation of the Uba1~ubiquitin thioester conjugate (Fig. 3a,b), but not formation of the Rad6~ubiquitin thioester conjugate resulting from transfer of the ubiquitin moiety from already formed Uba1~ubiquitin (Fig. 3d). The two most obvious possible mechanisms for inhibition of Uba1~ubiquitin thioester formation are that EGCG directly targets Uba1, possibly by binding to its active site, or that EGCG directly targets ubiquitin in such a way that it cannot be charged onto Uba1. The former possibility—that EGCG directly binds and thus inhibits Uba1—is far more likely. We determined that EGCG binds Uba1 by ligand-detected ^1^H NMR methods (Fig. 4) with an approximated *K*_d_ in a range similar to the IC_50_ values for inhibition of PCNA ubiquitination and Uba1–ubiquitin thioester formation by EGCG, strongly suggesting that it is the direct binding of Uba1 that accounts for the inhibitory activity. Furthermore, preincubation of Uba1 alone with EGCG prior to addition of the other reaction components (ATP and ubiquitin) of the Uba1~ubiquitin thioester formation results in inhibition of the reaction, while preincubation of ubiquitin alone with EGCG does not (Fig. 3a). This implies that ATP and/or ubiquitin compete effectively with EGCG for binding to Uba1, and so EGCG may bind the ATP- or ubiquitin-binding site of Uba1.

EGCG has been previously shown to inhibit the autoubiquitination activity of the E3 ubiquitin ligase TRAF6^43,44^, although, while EGCG does appear to bind TRAF6^44^, the reduced TRAF6 ubiquitination observed with treatment in the published functional experiments could also be the result of upstream inhibition of Uba1 and not necessarily TRAF6 directly. TRAF6, like Rad18, contains a so-called really interesting new gene (RING) zinc finger domain, thought to be involved in protein–protein interactions. We have no evidence thus far that EGCG directly inhibits Rad18. There are over 600 E3 ligases encoded by the human genome, the vast majority of them of the RING type, which display great structural diversity, particularly outside of the RING domain itself and in the substrate-binding domain (reviewed in^45,46^). Our results instead suggest that EGCG inhibits ubiquitination at the level of Uba1. It is worth noting, however, that EGCG and other catechin galloyl esters like ECG also obstruct another component of the overall ubiquitin–proteasome system: they appear to acylate and irreversibly inhibit the proteasome^47,48^, with a mechanism similar to that first described for the *Streptomyces* metabolite lactacystin, whose elucidation helped establish that the proteasome is an N-terminal threonine protease^49^.

Uba1 has been proposed as a target for treatment of cancers and other pathological states linked to detrimental levels of ubiquitin–proteasome activity and other ubiquitination-dependent activities (reviewed in^31–33^). The overall approach of intervening therapeutically in the ubiquitin–proteasome pathway has been most dramatically validated by the success of the proteasome inhibitor bortezomib (Velcade) in the treatment of multiple myeloma. However, investigations to validate Uba1 itself as a drug target are still few in number. Notably, the pyrazolidines PYR-41^50^ and PYZD-4409^51^ and the adenosine sulfamate TAK-243 (formerly known as MLN7243)^52^ have been found to inhibit Uba1 and appear to have promising therapeutic potential. TAK-243 is a potent mechanism-based inhibitor of the charging of Uba1 with ubiquitin that acts by forming a stable covalent adduct with the C-terminal carboxylate of ubiquitin, its direct target. TAK-243 and related reactive adenosine derivatives also likewise indirectly inhibit other ubiquitin-like protein-activating enzymes to varying degrees^52–55^. In contrast, although considerably less potent than TAK-243 (or EGCG and myricetin), PYR-41 appears to directly inactivate Uba1 itself rather than ubiquitin^50^, as does EGCG. TAK-243 and PYR-41 also differ in some of their biological effects^56^. A few other synthetic molecules^57,58^, natural products^59–61^, and modified ubiquitin derivatives^62^ have also been shown to inhibit Uba1.

In addition to being the likely EGCG target relevant to ubiquitination, Uba1 may account for part of EGCG’s overall cellular effects, given the broad importance of ubiquitination in cells. We found that overexpression of Uba1 in cells reduces EGCG’s cytostatic and cytotoxic effects (Fig. 5b), consistent with Uba1 being a major intracellular EGCG-binding protein in the cell. This is most likely the result of titration of free EGCG by higher Uba1 concentrations in the cell with overexpression. Overexpression of a compound-binding protein and evaluating whether sensitivity of cells is reduced to the compound’s effects or not has become an established approach to the confirmation of biological relevance of that putative target in explaining the compound’s cellular effects^63–65^. We also observed such a protective effect with overexpression of ubiquitin (Fig. 5b), further suggesting that the ubiquitination pathway is being targeted in the cell, and this is also still consistent with the hypothesis that Uba1 is the direct target of EGCG relevant to ubiquitination. The productive reaction in formation of the Uba1~ubiquitin thioester conjugate is trimolecular (involving Uba1 as covalent catalyst and ubiquitin and ATP as substrates), and overexpression of a binding partner of the direct target could also be protective if EGCG binds the target competitively with that additional component. The protective effects of Uba1 or ubiquitin overexpression are specific for EGCG and its biochemically active analogues, since neither of these manipulations affects sensitivity of cells to inactive analogues or to puromycin, a well-characterized protein synthesis inhibitor employed here as a representative unrelated cytotoxic agent (Fig. 5b).

Furthermore, we found that EGCG inhibited global ubiquitination in cells (Fig. 5c). A previous study has shown that EGCG treatment results in upregulation of the expression of the E3 ligase RNF216 and thus increased ubiquitination and degradation of one of its substrates, the Toll-like receptor 4^66^. However, this work was conducted in macrophages at a relatively low concentration of EGCG with long treatment duration, conditions that may preclude inhibition of Uba1 in the cells (particularly since, with a single treatment over longer time scales, EGCG would become extensively degraded and metabolized to simpler products), but which results in increased expression of RNF216 and so enhanced ubiquitination of its specific substrates.

EGCG and related polyphenols are interesting compounds with medicinally important promise, awareness of which may further encourage the healthful and civilized practice of green tea consumption—to quote the Japanese scholar Okakura Kakuzō^67^: “Tea began as a medicine and grew into a beverage.” While inhibition of Uba1 and ubiquitination may account for part of EGCG’s overall activity in cells, EGCG clearly inhibits various other cellular pathways as well, some no doubt involved in its aggregate health-beneficial effects. Pleiotropic activity profiles are not uncommon for bioactive small molecules, and often it is indeed the felicitous combination of multiple targets inhibited that is the basis of preventive or therapeutic efficacy of a drug. Nonetheless, SAR investigations may clear paths toward the development of more selective compounds with improved activity profiles and greater utility. While the “co-evolution” of natural products with proteins means that they intrinsically have favorable protein-binding properties, it can also present challenges in terms of selectivity between targets, as many bind conserved motifs in proteins. However, generation of structures derived from or inspired by specific natural products, synthetically or semisynthetically, could improve selectivity for one protein over another. Such efforts could be guided by comparative SAR studies across different systems.

We tested analogues of EGCG differing in constitution and stereochemistry, and an informative multi-assay SAR picture has emerged (Figs 2, 3 and 5 and Table 1; summarized with structures in Fig. 6). The EGCG-related molecules showed a divergence of different SAR profiles, with the comparative activity patterns falling into the following groupings: (1) catechin gallates (EGCG and ECG), which show very good correspondence between activity profiles in the different assays, being inhibitory in the *in vitro* biochemical (PCNA ubiquitination and Uba1~ubiquitin thioester formation) and cellular assays (cell viability—with negative effects mitigated by overexpression of Uba1 or ubiquitin); (2) simple unesterified catechins, which display no (EC and Cat) or weak (EGC) activity in the assays; (3) longer-chain alkyl gallates (OG and DG), which are active in all the assays, with their cytotoxic effects being mitigated in cells by overexpression of Uba1 or ubiquitin (while both the short-chain alkyl gallate PG and free GA itself are inactive in the biochemical assays, and their negative effects on cell viability are unaffected by Uba1 or ubiquitin overexpression); or (4) Myr and DM, which have potent (Myr) or weak (DM) activity in the biochemical assays and only weak activity against cell survival, with Uba1 or ubiquitin overexpression reducing the sensitivity of cells to these compounds.

All the compounds that exhibited activity in the *in vitro* PCNA ubiquitination and Uba1~ubiquitin thioester formation experiments also had cytostatic/cytotoxic effects that were mitigated with overexpression of Uba1, ubiquitin, or both (Fig. 5b). This suggests that all the bioactive compounds were also binding Uba1 and/or ubiquitin in the cell and that inhibition of ubiquitination at that level is relevant to the physiological activity of all these compounds. However, it is possible and indeed likely that the different structural classes of bioactive compounds, while each targeting some site on Uba1 and/or ubiquitin itself are also mechanistically distinct.

The potent inhibitory activity of the catechin gallates against the ubiquitination cascade appears to require a composite structure of both catechin and galloyl moieties. EGCG is an ester of EGC and GA, while ECG is an ester of EC and GA. Free EGC itself has weak activity in the biochemical assays (Figs 2b and 3b), while free GA is inactive, as is free EC (Figs 2c and 3c). It is known that galloylation also strongly influences various other bioactivities of catechins and other natural products (reviewed in^68^). Furthermore, the 5′-OH of the B ring on the catechin skeleton of EGCG seems to play a role in activity, possibly through hydrogen bonding with Uba1, since ECG, which lacks this hydroxyl group, has slightly diminished biochemical activity (see Fig. 6). Similarly, free EGC by itself still possesses some activity, while EC does not, again demonstrating the significance of the B-ring 5′-OH.

The mechanism of action of the longer-chain alkyl gallates (OG and DG) in inhibiting ubiquitination may be distinct from that of the catechin gallates and Myr (and its formal hydrogenation product DM). OG and DG, both esters of *n*-alkanols (of length C8 and C12, respectively) and GA, are amphiphiles with a polar “head” and straight-chain hydrophobic “tail.” Even though the fact that they are active against the PCNA ubiquitination cascade at relatively low concentrations suggests some element of specific recognition involved, they may have detergent-like local-denaturing effects upon binding, consistent with the increasing inhibitory activity in the biochemical assays with longer alkyl chain, with the order of potency being DG > OG ≫ PG (Figs 2b,c, 3b,c and Table 1). They exhibit high inherent variability in the biochemical experiments, and DG, in particular, displays large divergence in potency between the PCNA ubiquitination and Uba1~ubiquitin thioester formation assays (Table 1). These results suggest that, unlike the catechin gallates and Myr, the alkyl gallates may weakly inhibit multiple components of the cascade in the reconstituted PCNA ubiquitination system and without a single specific recognition interaction, with less of the more straightforward drug-like behavior against Uba1 exhibited by the other bioactive compound classes.

Myr likely also has a unique mechanism of action. Myr and DM do not possess a galloyl moiety, as do the catechin gallates and alkyl gallates. Myr potently inhibits thioester formation whether preincubated with Uba1 alone or ubiquitin alone before addition of other reaction components, whereas EGCG inhibits when preincubated with Uba1 but not ubiquitin (Fig. 3a). These results suggest either that Myr binds Uba1 non-competitively with ATP and ubiquitin or that Myr binds ubiquitin directly, in either case inhibiting formation of the Uba1~ubiquitin thioester conjugate by a mechanism that is distinct from EGCG. Furthermore, unlike all the other molecules examined in this study, Myr and DM are both ketone carbonyl compounds, and this may be key to their activity.

The strong activity of Myr may be tied to its Michael acceptor (α,β-unsaturated carbonyl) functionality, a potentially reactive electrophile susceptible to attack by nucleophilic groups on proteins and other molecules. In the Michael addition reaction, a nucleophile adds to the electrophilic β carbon of the α,β-unsaturated carbonyl. In such a mechanism for Myr, the carbon labeled “β” in Fig. 6 would be the site of attack by a nucleophile on Uba1 (such as the side-chain thiol of its active-site cysteine, normally involved in reaction with ubiquitin to form the Uba1~ubiquitin thioester adduct). DM, which lacks an α,β-unsaturated carbonyl, is much more weakly active against ubiquitination and Uba1~ubiquitin thioester formation than Myr, consistent with such a mechanism. Since the Michael reaction is often irreversible, we tested whether Myr irreversibly inhibits Uba1 and/or ubiquitin function or not. We found that Myr, like EGCG, appears to inhibit ubiquitination in a reversible manner (Fig. 2d). While, on the face of it, this may make such a mechanism seem less probable, a covalent though reversible reaction is still not unlikely to be involved. The electronic and steric effects of the substitution pattern on the α and β carbons, as well as the nature of the incoming nucleophile on the target, may make Michael addition to Myr reversible through β elimination. Indeed, such chemical parameters are known to influence reactivity and reversibility in Michael acceptors, with numerous examples of reversible bioactive Michael acceptor-containing compounds in the literature (reviewed in^69,70^).

The Michael reaction is a 1,4-addition (conjugate addition) reaction, where the incoming nucleophile adds to the β carbon of the α,β-unsaturated carbonyl. Another reaction typical of these systems is 1,2-addition (direct addition), where the nucleophile reacts with the carbonyl carbon instead of the β carbon. (Direct addition is, in fact, kinetically, though not thermodynamically, favored over conjugate addition.) It is generally reversible for weakly basic nucleophiles, such as thiols, alcohols, and amines, the most common nucleophiles present in proteins. Therefore, a reversible covalent protein–small molecule species could also be the result of a direct addition of a protein nucleophile to the ketone carbonyl on Myr, a reaction also be available to DM.

Both reversible Michael addition and direct addition are possibilities to explain why the α,β-unsaturated carbonyl appears important for activity in Myr, and these candidate mechanisms are not mutually exclusive. In contrast, in DM, only direct addition to the ketone is possible, which could conceivably explain its weak residual activity. This is consistent with the observation that Cat, which differs from DM only in lacking the ketone, is entirely inactive. Thus, both conjugate and direct addition may be involved in Myr’s inhibitory activity, with conjugate addition likely playing the larger role. However, it is not possible to exclude at this point purely structural explanations that do not involve any covalent reaction. The compounds differ geometrically in addition to electronically: Myr possesses a planar α,β-unsaturated system, while DM has tetrahedral α and β centers. Therefore, Myr’s mechanism of action could involve reversible covalent reaction(s), non-covalent inhibitory interaction between small molecule and protein, or a combination of these non-mutually exclusive mechanisms.

Green tea polyphenols, particularly EGCG, have widely acknowledged potential as preventive or therapeutic agents against a range of cancers and other disease states. Inhibition of Uba1 and ubiquitination, recognized as promising loci for therapeutic intervention in many of the same diseases, may account for part of EGCG’s overall biological effects, and so the present work may help lead to a better understanding EGCG’s health-beneficial properties. Moreover, the fact that EGCG and its analogues fall into distinct mechanistic subclasses that are rather cleanly correlated to structure opens up exciting possibilities for future research. Each series of bioactive small molecule identified represents a different first-in-class type of chemical modulator of ubiquitination, a hierarchical cascade of reactions that has drawn great interest from both the basic research and drug discovery communities. Improved specificity and selectivity may arise from assessment of further natural, semisynthetic, or synthetic congeners of the pharmacophore types delineated in the present study, yielding valuable therapeutic drug leads and tools from these different structural and mechanistic sets of molecules.

## Methods

### Materials

His-tagged human Uba1 in the pET3a bacterial expression vector^71^ (Addgene Cat#63571) was expressed in the *E*. *coli* strain BL21(DE3) and purified with Ni-NTA agarose beads (Macherey-Nagel). Purified *S*. *cerevisiae* RFC (all 5 subunits coexpressed in the *E*. *coli* strain BL21-Gold(DE3)), human Rad6B–Rad18 complex (coexpressed as in *S*. *cerevisiae* strain BJ5464), FLAG-tagged human Rad6B, FLAG-tagged human PCNA, and glutathione-*S*-transferase (GST)-tagged human ubiquitin were prepared as previously described^34,35,72,73^. Proteins that were expressed as GST fusions had the GST moiety removed with PreScission protease (GE Healthcare), with the exception of the GST-ubiquitin used in GST-based detection experiments. Concentrations of purified proteins were determined by measuring ultraviolet light absorbance at 280 nm in a spectrophotometer and applying the Beer-Lambert law with the calculated extinction coefficient for each protein.

Biotinylated human ubiquitin was purchased from Boston Biochemicals/R&D Systems (UB-570). Nicked DNA for PCNA loading was prepared by incubating purified pUC19 plasmid with Nt.BstNBI (New England Biolabs) at 55 °C overnight. Streptavidin donor and anti-FLAG acceptor AlphaScreen and AlphaLISA beads were purchased from PerkinElmer. Anti-FLAG M2 monoclonal antibody conjugated to horseradish peroxidase (HRP) was purchased from Sigma-Aldrich, HRP-conjugated anti-GST antibody conjugate was from GE Healthcare, and anti-DNA polymerase δ catalytic subunit antibody (A-9) and anti-β-tubulin monoclonal antibody (D-10) was from Santa Cruz Biotechnology.

EGCG and related molecules utilized in this study were from an in-house compound collection at Avicor or purchased from Selleck Chemicals or Adooq Bioscience. All small molecules were dissolved in dimethyl sulfoxide (DMSO) to make concentrated stock solutions and further serially diluted in DMSO to appropriate concentrations, as needed, before use in experiments. Compounds, both as solids and short-term DMSO stock solutions, were stored desiccated until use. Periodic analysis by liquid chromatography–mass spectrometry confirmed stable structural integrity of all of the compounds, without decomposition, both as solids and in DMSO.

### PCNA ubiquitination

The PCNA loading and ubiquitination cascade was reconstituted in 96-well white round-bottom polypropylene plates (Greiner) in a buffer consisting of 40 mM Tris-HCl, pH 7.5, 8 mM MgCl_2_, and 10% glycerol with 2.5 nM nicked circular pUC19, 50 nM RFC, 50 nM Uba1, 250 nM Rad6–Rad18 dimer, 50 nM FLAG-PCNA, and 250 nM biotin-ubiquitin (final concentrations). Compounds were added to the samples, with preincubation for 15 min at 25 °C before addition of ATP to a final concentration of 2 mM (to initiate the reaction cascade), followed by incubation for 2 h at 25 °C. The reactions were then terminated by addition of EDTA to 20 mM for Alpha assays or Laemmli sample buffer for Western blot analyses. For Alpha assays, reaction mixtures were diluted in buffer containing streptavidin-conjugated donor beads and anti-FLAG antibody-conjugated acceptor beads in opaque white microplates under low-light conditions, as recommended by the Alpha bead manufacturer, PerkinElmer. The plates were read on a Tecan Spark microplate reader at 23 °C. For Western blot analyses, FLAG-PCNA was detected with anti-FLAG antibody, and quantitations from the Western blot images was performed with NIH ImageJ software^74^ and then analyzed with GraphPad Prism software.

For reversibility, 7.5 μM Uba1 was incubated with 30 µM of EGCG or Myr (or DMSO carrier solvent alone as a control) for 2 h at 25 °C. Samples were then transferred to YM-30 Microcon centrifugal concentrators (Merck Millipore), diluted in buffer (40 mM Tris-HCl, pH 7.5, 8 mM MgCl_2_, and 10% glycerol), then serially centrifuged with additions of buffer to the remaining retentate between each spin, for final dilutions of compounds to <0.05% of original concentrations. The resulting samples were then tested in PCNA ubiquitination reactions.

### Uba1~ubiquitin thioester formation

The assay was carried out in a buffer consisting of 40 mM Tris-HCl, pH 7.5, 8 mM MgCl_2_, and 10% glycerol with final concentrations of Uba1 and GST-ubiquitin of 150 nM and 450 nM, respectively. Following preincubation of Uba1 alone with compounds for 15 min at 25 °C, GST-ubiquitin and ATP (the latter to 2 mM) were added, and the samples were incubated for 2 h at 25 °C. Laemmli sample buffer with or without dithiothreitol (DTT) as reducing agent was added into each reaction, and samples were resolved on 8% sodium dodecyl sulfate (SDS)-polyacrylamide gels, with visualization by silver staining or Western blot analysis with anti-GST antibody. For silver staining, gels were fixed in 40% methanol and 10% acetic acid for 20 min, sensitized for 1 min with 0.02% sodium thiosulfate, stained for 10 min with 0.1% silver nitrate in 0.01% formaldehyde, and developed in 3% sodium carbonate and 0.05% formaldehyde. Quantitative determination of the extent of Uba1~ubiquitin thioester formation was carried out from scanned images of silver-stained gels of reaction samples without DTT with NIH ImageJ software^74^ followed by analysis with GraphPad Prism software. GST-ubiquitin rather than untagged ubiquitin was used because the greater distance separation of free Uba1 and Uba1~ubiquitin bands on the non-reducing SDS–polyacrylamide gels allowed for clearer resolution of the Uba1~ubiquitin thioester conjugate, while still displaying similar kinetics and levels of thioester formation.

### Rad6~ubiquitin thioester formation

The overall reaction was conducted in two separate steps: initial reaction of Uba1 with ubiquitin in the absence of compound, then mixing with Rad6 with compound to evaluate transfer of ubiquitin from already formed Uba1~ubiquitin to Rad6. The first reaction was conducted with 300 nM Uba1, 1 μM GST-ubiquitin, and 2 mM ATP in a buffer of 40 mM Tris-HCl, pH 7.5, 8 mM MgCl_2_, and 10% glycerol for 30 min at 25 °C to allow for charging of Uba1 with GST-ubiquitin incubations. Separately, Rad6 (1 μM) was preincubated with EGCG for 15 min at 25 °C, also in a buffer of 40 mM Tris-HCl, pH 7.5, 8 mM MgCl_2_, and 10% glycerol, and then the precharged Uba1 from the first reaction was added as an equal volume to the compound-treated Rad6 samples (for final concentrations of Uba1, Rad6, and GST-ubiquitin of 150 nM, 500 nM, and 500 nM, respectively), with incubation of the mixture for another 15 min at 25 °C to allow for transfer of ubiquitin from Uba1 to Rad6. The samples were then subjected to SDS-polyacrylamide gel electrophoresis under non-reducing conditions, silver staining, and quantitation of scanned images, as described above for the silver-staining-based Uba1~ubiquitin thioester formation assay.

### NMR analysis of EGCG–Uba1 binding

NMR spectra were acquired with a Bruker Avance 600 MHz spectrometer equipped with a 5 mm z-gradient CP-TCI triple-resonance cryoprobe at 303 K. EGCG and Uba1 were dissolved in 20 mM phosphate-buffered saline at pH 7.4 (90% H_2_O, 10% D_2_O), containing 150 mM NaCl and 0.02% NaN_3_. In order to prevent EGCG oxidation, 1 mM tris(2-carboxyethyl)phosphine was added to the solutions. EGCG and Uba1 concentrations were 400 μM and 4 μM, respectively. All the spectra were acquired with excitation sculpting solvent suppression pulse scheme. In STD experiments, 40 equally spaced 50 ms Gaussian-shaped pulses were used for the selective saturation of the protein, thus the total saturation time was 2 s. The on-resonance irradiation and the off-resonance saturation frequency were set at 0.86 ppm and 40.0 ppm, respectively. A total of 2k scans were collected for each pseudo 2D experiment. 2D NOESY experiments were acquired with 128 increments and a mixing time of 100 ms. As a control, all the experiments were repeated for a sample containing EGCG at the same concentration but in the absence of the protein.

### Cell culture

For cellular experiments, HEK 293FT cells (a fast-growing SV40 large T antigen-transformed strain of HEK 293 cells designed for enhanced transgene expression in transient transfections) were grown in growth medium consisting of Dulbecco’s modified Eagle’s medium (DMEM; Sigma-Aldrich), supplemented with 10% fetal bovine serum (FBS; Gibco) in a humified cell culture incubator at 37 °C with 5% CO_2_.

### Cell survival

HEK 293FT cells were evaluated for survival following compound treatments by the Alamar Blue (resazurin) assay. Cells were plated onto 96-well plates at 4.8 × 10^4^ cells/well in 0.5% FBS-containing DMEM at 37 °C and 5% CO_2_. After 24 h, compounds were added. After 24-h treatment, resazurin was added to a final concentration of 0.12 mM. Following 4-h incubation, conversion to the resorufin product was measured (with excitation at 542 nm and emission at 590 nm) on a fluorescence plate reader.

### Cell transfections

HEK 293FT cells (1.5 × 10^6^) were plated onto 6-cm plates in 5 ml of DMEM with 10% FBS and allowed to grow for 24 h at 37 °C and 5% CO_2_. For transfections, 6 μg of plasmid DNA (empty vector containing FLAG-tag sequence only, FLAG-Uba1, or FLAG-ubiquitin) and 10 μl of Lipofectamine 2000 were used for each plate. The DNA and the reagent were diluted separately in 500 μl of Opti-MEM (Gibco), and each was mixed by vortexing. After 5-min incubation, the two tubes were combined and incubated for another 20 min. Prior to adding the transfection solution to the cells, the medium was replaced with 4 ml of Opti-MEM. The transfection solution was then added dropwise. After 3-h incubation, the medium was changed to 0.5% FBS-containing DMEM, and the cells were plated for cell survival experiments, as above.

### Cellular ubiquitination

HEK 293FT cells were plated in 6-well plates at 8 × 10^5^ cells/well in DMEM with 0.5% FBS. After 24 h at 37 °C and 5% CO_2_, EGCG was added; then 30 min later, MG132 was added to a concentration of 50 μM, with incubation for another 30 min (for a total of 1-h EGCG treatment). Cells were harvested, and whole-cell lysates were prepared in RIPA lysis buffer containing protease inhibitor cocktail (SIGMA*FAST* from Sigma-Aldrich) and sonicated. Equivalent loadings of total protein for each treatment, as determined by Bradford assay of each extract sample, were subjected to electrophoresis on 8% SDS-polyacrylamide gels and Western blot analysis, probed with anti-ubiquitin antibody, then stripped and reprobed with anti-β-tubulin antibody.

### Statistical analysis

Curve fitting and statistical analysis were performed with GraphPad Prism 8.0–8.2 software. Quantitative data in graphs are presented as mean and standard deviation (SD).
